# Molecular Clock of Neutral Mutations in a Fitness-Increasing Evolutionary Process

**DOI:** 10.1371/journal.pgen.1005392

**Published:** 2015-07-15

**Authors:** Toshihiko Kishimoto, Bei-Wen Ying, Saburo Tsuru, Leo Iijima, Shingo Suzuki, Tomomi Hashimoto, Ayana Oyake, Hisaka Kobayashi, Yuki Someya, Dai Narisawa, Tetsuya Yomo

**Affiliations:** 1 Faculty of Science, Toho University, Funabashi, Chiba, Japan; 2 Faculty of Life and Environmental Sciences, University of Tsukuba, Tsukuba, Ibaraki, Japan; 3 Graduate School of Information Science and Technology, Osaka University, Suita, Osaka, Japan; 4 The Organization for the Promotion of Leading Graduate Schools, Nagoya University, Furo-cho, Chikusa-ku, Nagoya, Aichi, Japan; 5 QBiC, RIKEN, Suita, Osaka, Japan; 6 Graduate School of Frontier Sciences, Osaka University, Yamadaoka, Suita, Osaka, Japan; 7 Exploratory Research for Advanced Technology (ERATO), Japan Science and Technology Agency (JST), Suita, Osaka, Japan; National Institute of Genetics, JAPAN

## Abstract

The molecular clock of neutral mutations, which represents linear mutation fixation over generations, is theoretically explained by genetic drift in fitness-steady evolution or hitchhiking in adaptive evolution. The present study is the first experimental demonstration for the molecular clock of neutral mutations in a fitness-increasing evolutionary process. The dynamics of genome mutation fixation in the thermal adaptive evolution of *Escherichia coli* were evaluated in a prolonged evolution experiment in duplicated lineages. The cells from the continuously fitness-increasing evolutionary process were subjected to genome sequencing and analyzed at both the population and single-colony levels. Although the dynamics of genome mutation fixation were complicated by the combination of the stochastic appearance of adaptive mutations and clonal interference, the mutation fixation in the population was simply linear over generations. Each genome in the population accumulated 1.6 synonymous and 3.1 non-synonymous neutral mutations, on average, by the spontaneous mutation accumulation rate, while only a single genome in the population occasionally acquired an adaptive mutation. The neutral mutations that preexisted on the single genome hitchhiked on the domination of the adaptive mutation. The successive fixation processes of the 128 mutations demonstrated that hitchhiking and not genetic drift were responsible for the coincidence of the spontaneous mutation accumulation rate in the genome with the fixation rate of neutral mutations in the population. The molecular clock of neutral mutations to the fitness-increasing evolution suggests that the numerous neutral mutations observed in molecular phylogenetic trees may not always have been fixed in fitness-steady evolution but in adaptive evolution.

## Author Summary

Mutations that have little influence on biological function are referred to as neutral mutations and frequently appear in molecular phylogenetic analyses. The fixation of neutral mutations in populations has been attributed to genetic drift in fitness-steady evolutionary processes or hitchhiking in adaptive evolution. We examined the fitness-increasing evolution of *Escherichia coli* for thermal adaptation to observe the fixation dynamics of genome-wide mutations. In the adaptive evolution, all genomes in the population equally accumulated neutral mutations by replication errors. The infrequent occurrence of an adaptive mutation on one of the genomes by chance resulted in the fixation of the neutral mutations that had pre-accumulated in the same genome by hitchhiking. Via successive hitchhiking events, the neutral mutations were fixed in the population linearly over generations at the same rate as the spontaneous mutation accumulation rate in the genome. The molecular clock of neutral mutations thus functions even in adaptive evolution. The evolutionary period characterized by the accumulation of numerous neutral mutations observed in molecular phylogenetic trees may not be specific to neutral evolution but may occur in adaptive evolution as well.

## Introduction

The dynamics of adaptive evolution are more intricate than a simple sum of mutation and selection due to the entanglement of several evolutionary events [1], which include rare adaptive mutations [2,3,4,5,6,7,8], epistasis [9,10,11] and hitchhiking [12,13,14] at the genome level and clonal interference [15], frequency-dependent selection [16] and genetic drift [17] at the population level. Recent genomic sequencing analyses of yeast evolution experiment have confirmed that the rise and fall of adaptive genotypes are complicated due to the concurrence of hitchhiking and clonal interference [14].

Despite these recently uncovered complicated dynamics, a constant mutation fixation rate in a population is considered a simple rule in phylogenetic analysis. Based on fossil records, the molecular clock, which was first proposed in the 1960s, suggests that mutations accumulate over time [18]. Intensive studies largely developed the concept of molecular clock for broader applications [19,20,21]. Although the constancy of the mutation fixation rate may vary to some extent, the molecular clock has become a simple tool for evolutionary researchers to convert mutational differences into phylogenetic trees to trace past evolutionary events [19,22].

There are two possible mechanisms for the molecular clock. The first mechanism is the genetic drift of neutral mutations, also known as neutral evolution [23]. Mutations with no fitness contribution can drift to the majority by stochastic effects due to a limited population size. Because mutations occur at neutral sites at the spontaneous mutation accumulation rate and propagate equally, the mutational difference among independent populations derived from a common ancestor increases over generations in a clockwise manner at the spontaneous mutation accumulation rate. In selective environments, neutral mutations drifting toward fixation are swept out by adaptive mutations. The other mechanism is hitchhiking, in which neutral mutations accumulating on single genomes are fixed through the propagation of adaptive mutations that occurred last on the same genomes. No matter which of the genomes in a population gain an adaptive mutation, the number of neutral mutations that take hitchhiking does not vary greatly because all the genomes accumulate neutral mutations equally at the same spontaneous mutation accumulation rate, setting the molecular clock for neutral mutations [24,25,26] Thus, the fixation rate of neutral mutations by hitchhiking is expected to be identical to the spontaneous mutation accumulation rate, which is the same as the fixation rate by genetic drift. The main difference between the two mechanisms in neutral mutation fixation is the time scale. The mutation fixation that mediated by genetic drift requires much longer time than that mediated by hitchhiking. Theoretically, the number of generations is close to the population size in genetic drift, but is as small as the inverse of selection coefficient in hitchhiking [27].

While both genetic drift and hitchhiking occur in laboratory population dynamics, direct evidence that these mechanisms fix neutral mutations in proportion to the number of generations has not been obtained. Pioneering experimental population dynamics studies on fruit fly demonstrated that genetic drift fixes neutral alleles [28], and many studies have demonstrated that a substantial fraction of the mutations found in phylogenetic trees and in natural populations are attributable to fixation by genetic drift [27]. However, the process by which neutral mutations are successively fixed by genetic drift has not directly been observed. While hitchhiking has been observed in experimental populations [12,13,14], experimental evidence supporting a role of successive occurrences of hitchhiking in the linear fixation of neutral mutations over generations is limited. If hitchhiking fixes neutral mutations at a rate that is equal to the spontaneous mutation accumulation rate, the concept of a molecular clock could be extended to fitness-increasing evolution, which is not covered by the neutral theory of molecular evolution of Kimura.

Here, we report the repeated appearance of hitchhiking in a severe selective environment, leading to clock-like molecular evolution of neutral mutations. We previously conducted an evolution experiment in which *E*. *coli* was thermally adapted to 44.8°C [29]. Genome-wide analysis over the course of this evolution revealed that the majority of the accumulated mutations shifted from a positive to neutral fitness contribution. In the present study, we duplicated and prolonged the previous evolution experiment for further thermal adaptation to 46°C. The dynamic process of the genome-wide mutation fixation between the generation of the previous evolution, at which the fitness contribution of mutations shifted from positive to neutral, to the final generations of the duplicated evolutionary lines revealed that hitchhiking was responsible for the constant fixation rate of neutral mutations, even though the population dynamics of the genotypes were complicated due to the stochastic concurrence of adaptive mutations and clonal interference. The possibility that at least some of the neutral mutations that accumulated in the past were fixed by hitchhiking in adaptive evolution is discussed.

## Results

### Diversification in thermal adaptation accompanied by differentiation in genomic mutation fixation

**Fig 1:**
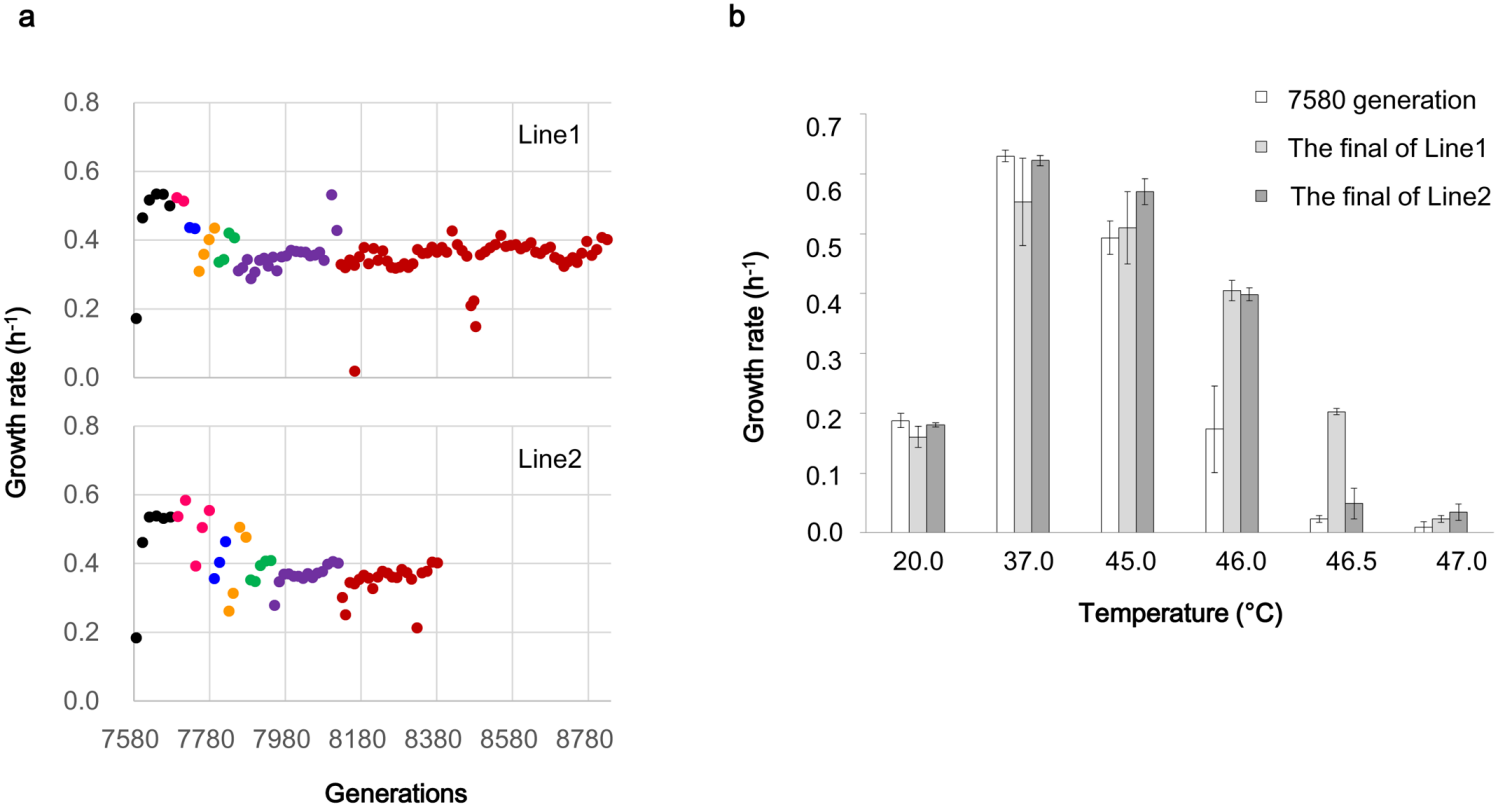
Growth rate changes in the evolution for thermal adaptation. The duplicated Line1 and Line2 from the final population at generation 7580 were passaged daily. The daily exponential growth rates at various temperatures were calculated based on the absorbance at 600 nm as described in the Materials and Methods section. a) Each color indicates the growth rates of the bacterial cells at 44.8°C (black), 45.0°C (pink), 45.2°C (blue), 45.4°C (orange), 45.6°C (green), 45.8°C (purple), and 46.0°C (red). The cell populations that were finally acquired at 46.0°C were subjected to genome sequencing analysis. b) The populations at generation 7580 (white) and the final populations of Line1 (gray) and Line2 (dark gray) were cultured to observe the growth rates at 20.0°C, 37.0°C, 45.0°C, 46.0°C, 46.5°C and 47.0°C. The average and standard errors were obtained by repeated cultures (n = 5–6).

To investigate the dynamics of genomic molecular evolution in a selective environment, we prolonged the previous thermal evolution experiment in which an *E*. *coli* cell population evolved to grow exponentially at 44.8°C [29]. The final population at 7580 generations was duplicated (Line1 and Line2) and challenged at the 0.2°C increment (Fig 1a). The increment was repeated whenever the exponential growth rate exceeded 0.4/h for two consecutive days until the cells of the duplicated lines were able to grow at 46.0°C. In terms of fitness increase, the selection pressure in the prolonged thermal adaptation evolution with the sequential increments of 0.2°C (from 7581 to 8382, and 7581 to 8829 generations) was equivalent to that in the previous report from 5212 to 7580 generation at 44.8°C [29], because the rates of fitness increase (the increase in growth rate per generation) were both at the order of 10^−4^, which was one order higher than the fitness increase (~10^−5^) observed in the nearly static phase at 37°C [29,30].

Although the reduction and recovery of the growth rate after every temperature increment were basically the same for the two lines, Line1 required more generations to reach 46.0°C than did Line2. The temperature dependence of the growth rate revealed that the final population of Line1 but not Line2 was able to grow at 0.5°C higher than the final temperature of 46.0°C (Fig 1b). The two lines, which originated from the same population and were subjected to the same rules for temperature increment, evolved to develop different thermal tolerances at different speeds.

Whole-genome mutation analysis of the final populations of Line1 and Line2 revealed difference in the number of accumulated mutations. A next-generation sequencer (Applied Biosystems SOLiD 3 system) was used to estimate the ratio of substituted bases to original bases in the two populations. We conducted Sanger sequencing to confirm possible single nucleotide polymorphism (SNP) candidates based on SOLiD data, except those SNPs in rRNA and tRNA. The ratios of SNPs determined from the SOLiD data were consistent with those determined by Sanger sequencing. The standard deviation of the ratio of SNPs was 6.2% for Sanger sequencing (S1 Fig). The proportion based on Sanger sequencing was used in the subsequent analysis for a precise and costless evaluation. In addition to the mutations that were identified in the final generation in the previous study, 82 new substitutions were confirmed, of which 39 and 20 were exclusive to Line1 and Line2, respectively (S1 Table). The greater number of mutations in Line1 compared to Line2 may be attributable to the greater number of generations of Line1 compared to Line2.

### Clustered fixation dynamics of the accumulated mutations on single genomes

**Fig 2:**
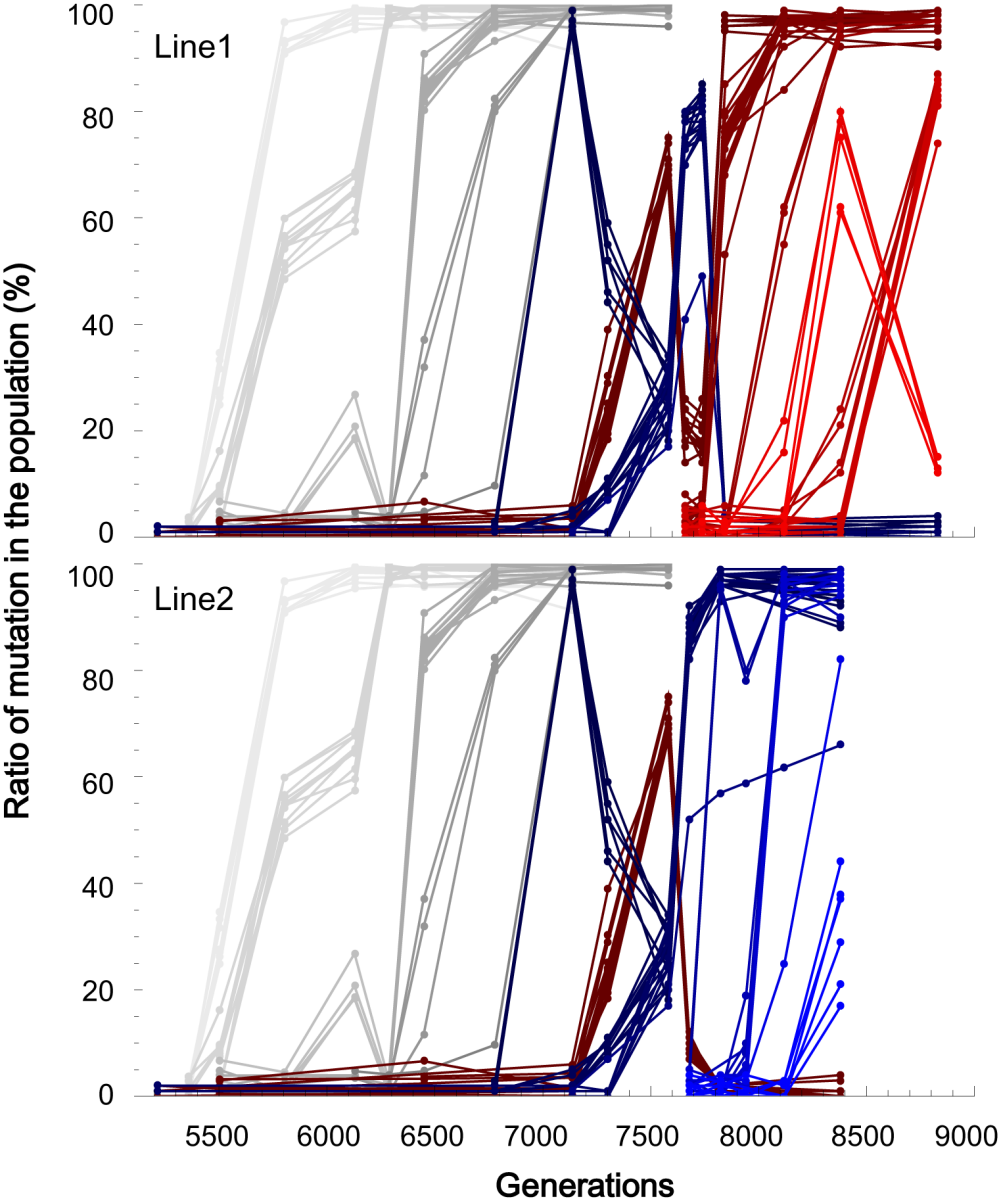
Temporal frequency change for each mutation. The frequency estimated by Sanger sequencing is plotted for each substituted base for Line1 (upper) and Line2 (bottom). The mutations that were inherited in Line1 were colored red, while those that were suppressed at the end of Line1 are colored in purple. The blue lines correspond to the mutations that were maintained at the end of Line2. The gray lines represent mutations that were inherited in both Line1 and Line2.

SNP temporal change from generation 5212 of the previous evolution experiment to the final generations of Line1 and Line2 revealed that the mutations were clustered in fixation dynamics, as predicted by hitchhiking. We determined the fixation processes of 46 mutations that appeared between generations 5212 and 7580 and the newly discovered 82 mutations with the estimated ratios of substituted bases to original bases by Sanger sequencing as previously described. The estimated frequencies (%) of the mutations for the intermediate and two final populations were listed in S2 Table. The 5212^th^ generation is the time point after which the spontaneous mutation accumulation rate accelerated. Several mutations simultaneously appeared over the detection limit of 5% for Sanger sequencing and proceeded together toward fixation (Fig 2).

Based on the concurrent frequency changes toward fixation, we clustered the 128 mutations into 22 clusters (S2 Table). To determine whether the mutations belonging to the same cluster were on single same genomes as predicted by hitchhiking, the eight mutations that belonged to the first cluster, A1, which appeared at generation 5504 and became the majority at generation 5802, were further examined by randomly isolating ten clones from the population at generation 5504. Among the ten clones, four possessed all eight mutations, while the other six clones did not exhibit mutations at the eight sites. Thus, these eight mutations were present on the same genome at generation 5504. The clone ratio of 4:6 is within the standard deviation from the average frequency of 29% over the eight mutations that was estimated from the peaks of Sanger sequencing at generation 5504 (S2 Table). Similarly, all 10 mutations (the fourth cluster, A4) that exhibited an average frequency of 84% at generation 6448 were observed at a clone ratio of 9:2 between clones with all 10 of the mutations and those with no mutations at the corresponding sites, and all three mutations (the fifth cluster, A5) that displayed an average frequency of 81% at generation 6780 were observed at a clone ratio of 7:3 between the clones possessing all or none of the three mutations. These results indicate that the clustering in the fixation dynamics is attributable to the accumulation of the mutations on the same genomes before becoming the majority in the population

### Hitchhiking of a nonsense mutation in *mutH* with other mutations

**Fig 3:**
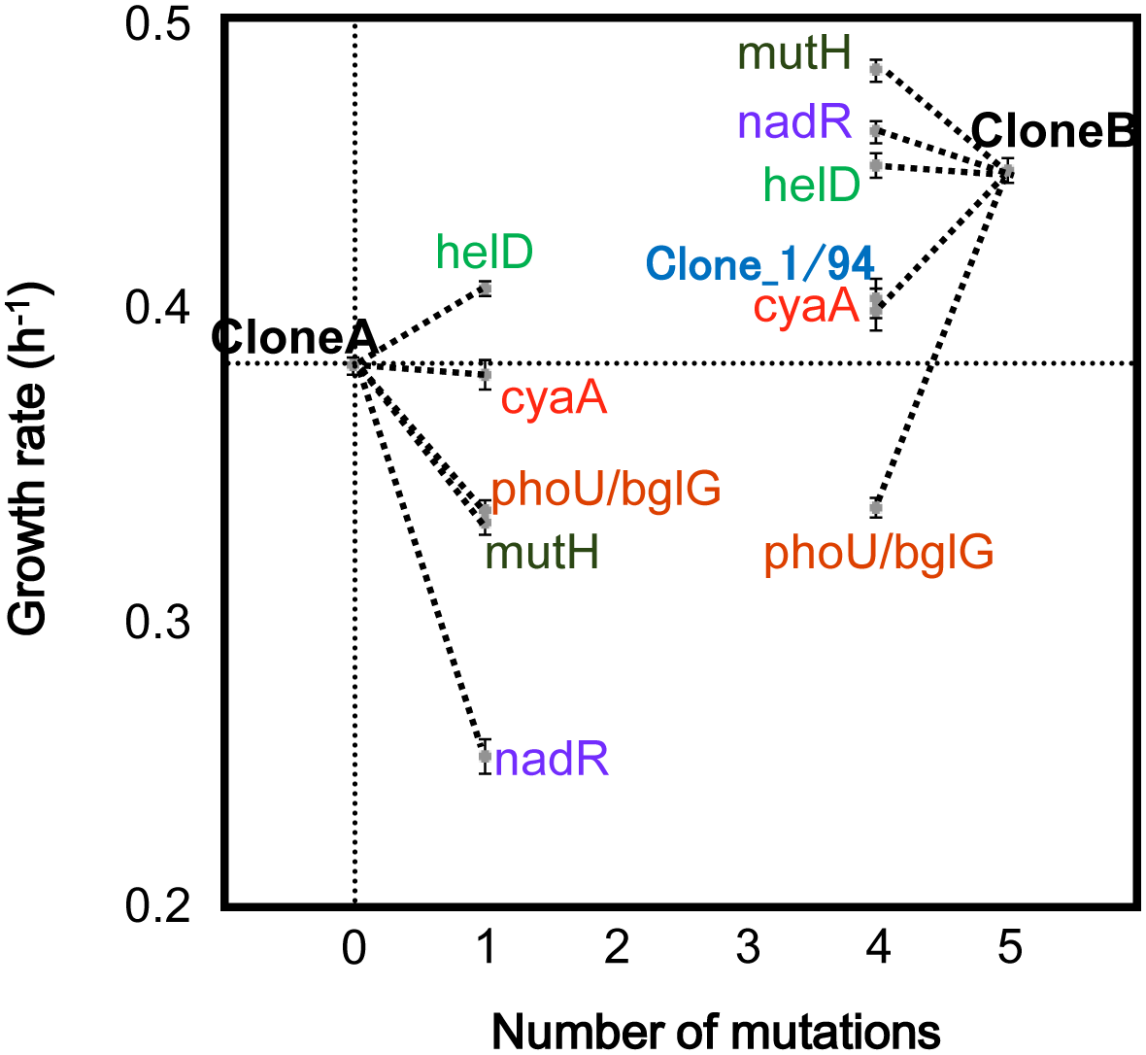
The effects of the five non-synonymous mutations in the first cluster on the growth rate. CloneA was isolated from the population at generation 5076, while CloneB was isolated from the population at generation 5504. Sanger sequencing confirmed that CloneB had all the mutations in the first cluster, while CloneA did not. The average and standard error for the growth rate at 44.8°C were determined by repeated growth experiments for each clone (n = 6–27). The five recombinant genotypes from Clone1 on one of the five mutations on *mutH*, *helD*, *cyaA*, *nadR* and *phoU/bglG* and the five recombinant genotypes back-mutated from CloneB on one of the five mutations are indicated by the names of the corresponding five genes. Clone_1/94, the clone found among randomly isolating 94 clones from the population at generation 5358 exhibited the same growth rate as the clone back-mutated on *cyaA* from CloneB.

The synthesis and growth rate analysis of the recombinant genotypes with the mutations in the first cluster, A1, which became the majority at generation 5802, indicated that the nonsense mutation in *mutH* hitchhiked on at least one of the other mutations in the same cluster. Mutations on mismatch repair genes that induce mutator phenotypes are fixed together with beneficial mutations that compensate for the genetic load due to high spontaneous mutation accumulation rates [13,31,32]. To determine if the *mutH* mutation hitchhiked on beneficial mutations, we genetically constructed ten *E*. *coli* strains (genomes), based on two different genetic backgrounds of CloneA and CloneB (Fig 3). The CloneA and CloneB genomes carried either none or all of the five mutations (four nonsynonymous and one non-coding region mutations, *i*.*e*., *mutH*, *helD*, *cyaA*, *nadR* and *phoU/bglG*) in the A1 cluster, respectively. Five genomes out of ten were derived from CloneA and were constructed by adding one of the five mutations to the genome before the fixation of the A1 cluster. The other five genomes derived from CloneB were constructed by back-mutating one of these five mutations to the genome holding the A1 cluster mutations.

Growth assay of these strains (genomes) revealed both the negative contribution of the *mutH* mutation and the epistasis between the mutations. The addition of the *mutH* mutation to CloneA reduced the growth (0.05 h^-1^ decrease) and its subtraction from CloneB rescued the growth (0.03 h^-1^ increase) (Fig 3), indicating that the negative contribution of the *mutH* mutation to the growth fitness was independent of the genetic background. The contribution of the other mutations to the growth fitness were somehow dependent on the genetic background. For instance, the growth rate of CloneB was higher (0.04 h^-1^ increase) than that of the *helD* mutation supplied CloneA (Fig 3). That is, the accumulation of the other four mutations positively contributed to the genome that carrying the *helD* mutation, nevertheless these four mutations were harmful to the genome without the *helD* mutation (CloneA) when occurred individually. The results strongly suggested the epistasis between these mutations. The growth deficiency caused by the *mutH* mutation might be also partially compensated by the other mutations on the same genome.

We confirmed this compensation by randomly isolating 94 clones from the population at generation 5358, the time point just before the A1 cluster rose above the lower detection limit (5%). Among the 94 clones, the *mutH* mutation appeared only on the single clone that was accompanied by the mutations in *helD*, *nadR* and *phoU/bglG* (Clone_1/94). Clone_1/94 harboring the four mutations (*mutH*, *helD*, *nadR* and *phoU/bglG)* showed the same growth rate as that of the genetically constructed clone of the same genetic background (*cyaA* back-mutation from CloneB), and showed slightly higher growth rate than that of CloneA. It indicated that the negative contribution of the *mutH* mutation to the growth fitness in the genetic background of CloneA was neutralized by the *helD*, *nadR* and *phoU/bglG* mutations (Fig 3). The growth fitness rescued by the three mutations might allow the cell (genome) carrying the *mutH* mutation to escape from extinction and to propagate till a substantial number of cell population, as the detected Clone_1/94. Because the growth rate of Clone_1/94 was lower than that of CloneB, a plausible order for the accumulation of the five mutations is *mutH*, (*helD*, *nadR* and *phoU/bglG)*, and *cyaA*. Although all of the combinations of the five mutations and three synonymous mutations in the A1 cluster should be constructed to examine all possible orders of mutation accumulation, it is reasonable to propose that the *mutH* non-synonymous mutation could take hitchhiking by neutralizing its negative fitness contribution with at least one of the other mutations in the first cluster A1.

### Concurrence of clonal interference and hitchhiking

**Fig 4:**
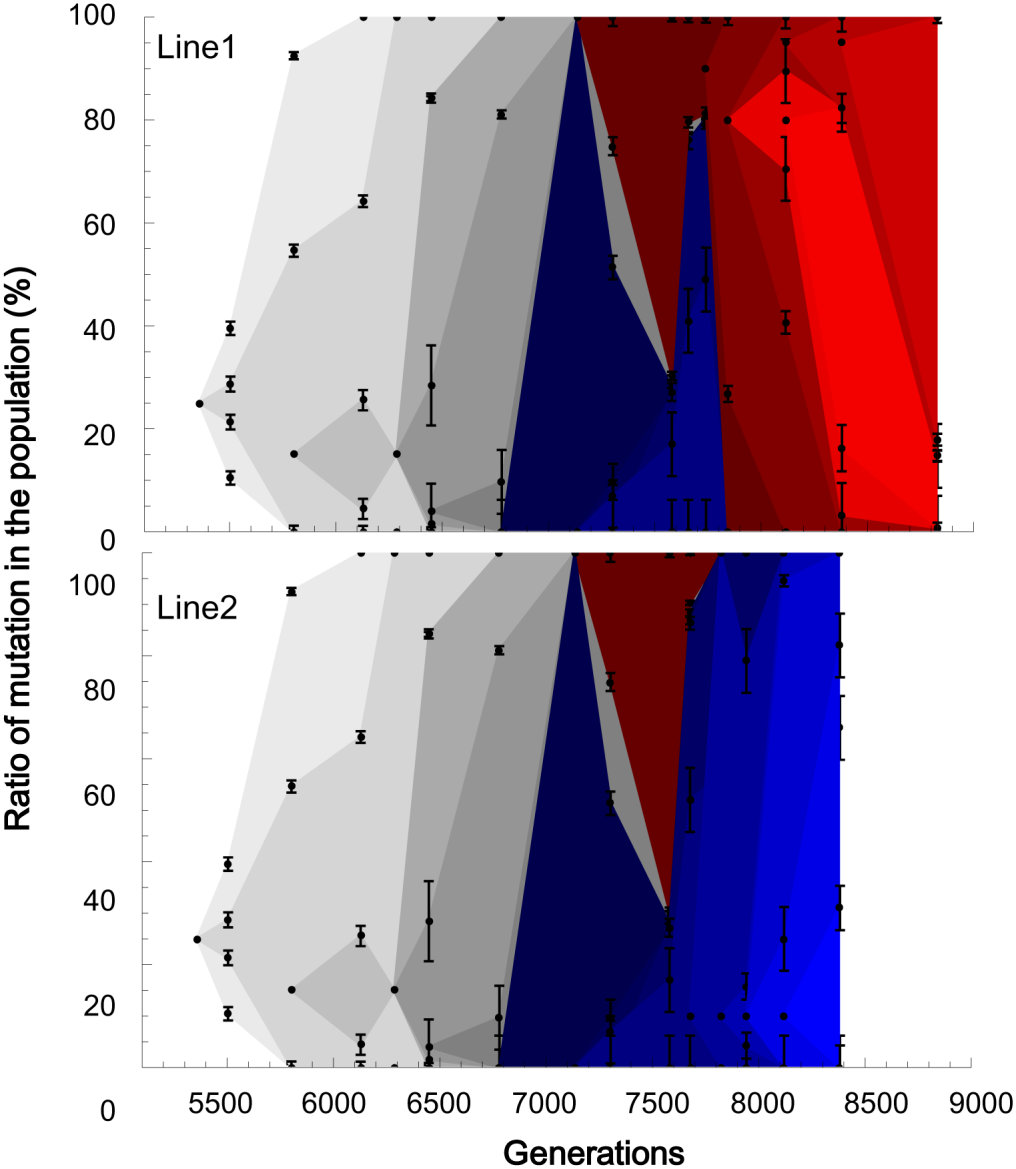
The fixation processes of the genomes. The frequency of each mutated genome at each generation was calculated as the average over the mutations in the same clusters. The bars are the standard errors. The standard error for the genomes only with single mutations was replaced with the standard deviation from Sanger sequencing (6.2%, S1 Fig). The spread for each genome began with an arbitrary height at one sampling point before the frequency went beyond the detection limit of 5%. A frequency higher than 95% was regarded as 100%. The gray scaled spreads correspond to the genome mutations that were inherited at the ends of Line1 and Line2. The red spreads correspond to the mutations that were inherited in Line1, while the blue spreads indicate the mutations that survived until the end of Line2.

The fixation process of the mutated genomes exhibited complicated dynamics due to the stochastic appearance of adaptive mutations and clonal interference. Theoretically, if adaptive mutations are introduced into populations excessively by a high spontaneous mutation rate or a large population size, these mutations will interfere with each other so that only a fraction of them will be fixed in populations [33]. Clonal interference has been reported in the experimental evolution of large populations [9,34,35,36]. In the experiential evolution of yeast, hitchhiking and clonal interference were simultaneously observed [14]. Consistent with these results for yeast, complicated dynamics were observed when we estimated the frequencies of the single-mutated genomes by averaging the frequencies over the mutations in the same clusters (Fig 4). Until generation 7134, the adaptive genomes stochastically appeared and simply increased their frequency, with the exception of a small fluctuation at generation 6128 for the genome corresponding to the A3 cluster. The fixation processes of the adaptive genomes overlapped as the frequency-increasing genomes gained other adaptive mutations before complete fixation. This overlap is a consequence of the accelerated spontaneous mutation accumulation rate. If adaptive mutations occur on different genomes, the adaptively mutated genomes may interfere with each other. In fact, from generation 7134 to 7580, the most dominant genome on Lineage Blue was interfered with by the other genome on Lineage Red. Lineage Blue survived to the end of Line2, while Lineage Red was inherited at the end of Line1.

Interestingly, only those genotypes that are 34 Hamming distances away from each other occupied the population at generation 7580 without any possible intermediate genomes (70% for the genome with the R1 cluster in Lineage Red, 28% for the genome with the B1~B3 cluster in Lineage Blue, see S2 Table). Generation 7580 is the time point at which the final population of the previous experimental evolution was duplicated into Line1 and Line2. By randomly isolating 20 clones at generation 7580, the coexistence of the two long-distanced genomes was confirmed; 14 clones belonged to the genotype on Red, while 6 clones were on Blue. The coexistence of the distanced genotypes can be attributed to the concurrence of hitchhiking and clonal interference. After generation 7580, further complication was observed in Line1: Lineage Blue, once pushed down by Red at generation 7580, returned to the majority at generation 7741 but became extinct with the domination of Red, which gained the cluster R2. A completely different fate was observed in Line2, where Lineage Blue ultimately eliminated Lineage Red. Thus, the genomic evolution demonstrated complicated population dynamics with the entanglement of the stochastic appearance of the adaptive mutations triggering hitchhiking and clonal interference.

### Molecular clock of the hitchhiking dynamics of neutral mutations

**Fig 5:**
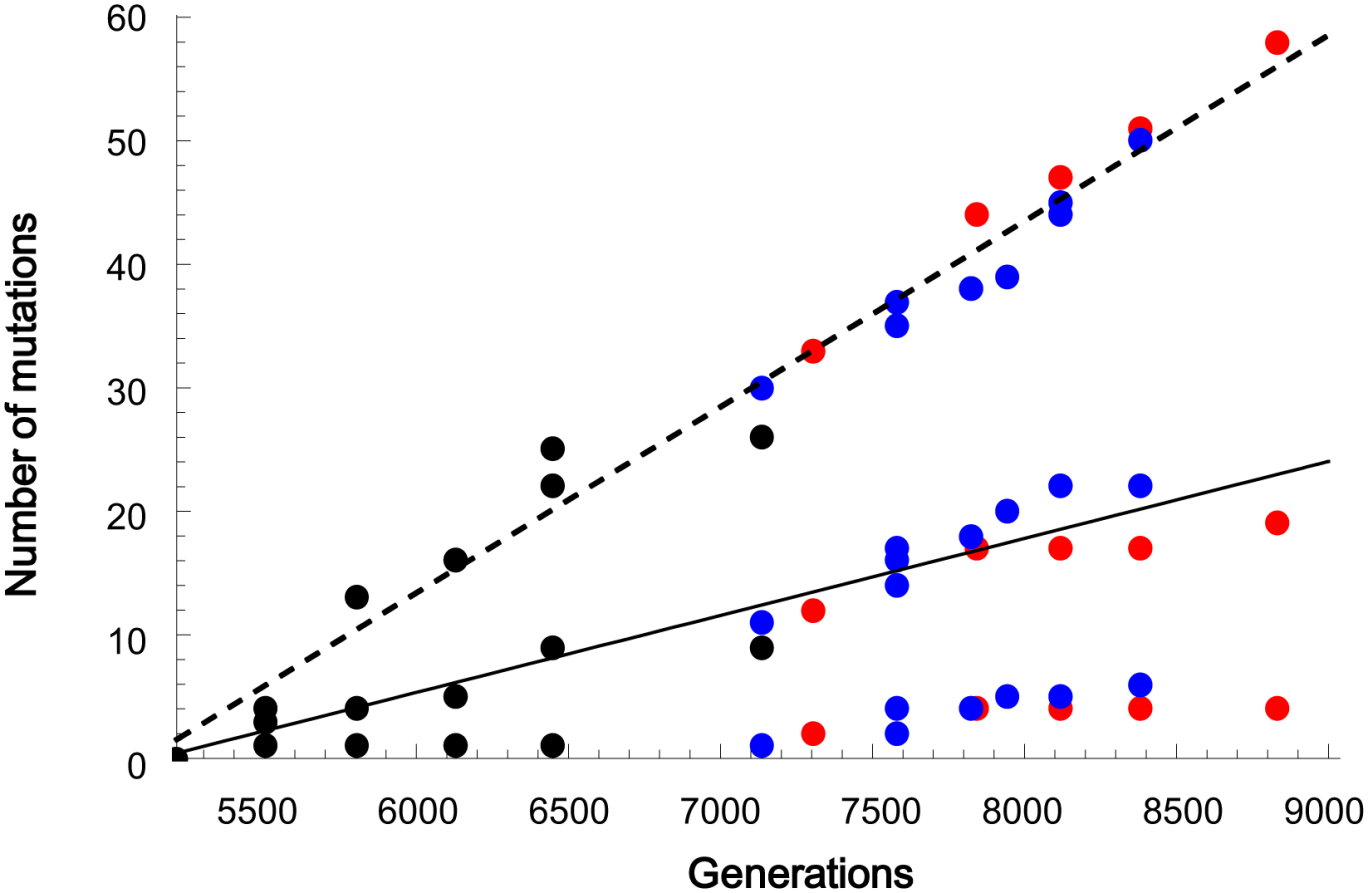
The linear accumulation of mutations over generations. The number of non-synonymous (top), synonymous (middle) and non-coding (bottom) mutations were plotted at the time point when the frequency of the mutated genome first became greater than 10%. The mutated genomes that appeared at the latter stage in Line1 and declined at the end, in Fig 3, which correspond to clusters R4 and R5 in S2 Table, were omitted to focus on only the lineages dominating at the ends of the two lines. The dashed and solid lines represent the regression lines for non-synonymous and synonymous mutations, respectively (n = 21, including no mutation at generation 5212, P < 10^−16^ for non-synonymous and P < 10^−10^ for synonymous).

Despite the complicated fixation dynamics of genomic mutation, the molecular evolution rate exhibited a simple clock-like constancy. Synonymous and non-synonymous mutations accumulated monotonously over generations (synonymous and non-synonymous mutations are indicated by middle dots and top dots, respectively, while mutations in non-coding regions are indicated by the bottom dots in Fig 5). Because each of the hitchhiking events propagated a single genome with various numbers of non-deleterious mutations at various rates, the data points deviated to some extent from lines of constant slope. The fixation rates in Line1 and Line2 were approximately equal to each other, with the exception of the specific data points for the synonymous mutations in Linkage Red and Blue after generation 7134 (Lineage Blue exhibited a slightly higher rate than Red, with n = 9 and 5, *P*<0.05). Even with these different population dynamics, the molecular clock rates of the two lineages were approximately the same order.

The synonymous mutations accumulated at a rate of the same order as the spontaneous mutation rate. By dividing the slope for the unified data of Line1 and Line2 by the synonymous nucleotide sites of 0.96 Mbp of *E*. *coli*, we estimated the fixation rate of synonymous mutations as 0.7×10^−8^ per generation per site. The rate is on the same order as the previously reported accelerated spontaneous mutation accumulation rate of 1×10^−8^ [29]. Because the synonymous mutations are close to neutral in fitness contribution, hitchhiking in the evolution experiment allowed neutral mutations to be fixed at the same rate as if genetic drift were involved. Because every genome in the population accumulated neutral mutations at the spontaneous mutation accumulation rate, regardless of which genomes were propagated by hitchhiking, the expected fixation rate of neutral mutations should be equal to the spontaneous mutation accumulation rate as theoretically discussed [25,26]. This is the first experimental demonstration of the molecular clock with the same rate as the spontaneous mutation accumulation rate.

Non-synonymous mutations also accumulated at a constant rate, as expected for hitchhiking. Theoretically, strong hitchhiking generated near-neutral fixation probabilities for mutations, although the mutations contributed to the fitness of varied magnitudes [24,37]. In the regimes of emergent neutrality, beneficial and deleterious non-synonymous mutations might affect each other and average out their individual fitness to be nearly neutral. Here, we assumed for simplicity’s sake that only a single non-synonymous mutation that occurred on a genome immediately before its propagation was adaptive, while the other non-synonymous mutations that pre-accumulated on the same genome, each of which could be positive or negative in fitness contribution to some extent, were averaged to be neutral. In fact, complementation of the fitness contribution among the non-synonymous mutations on the same genome was observed in the hitchhiking of the *mutH* mutation as described above. Based on this assumption, among the 82 non-synonymous mutations fixed through 20 independent propagation events (excluding, for simplicity’s sake, the mutations of cluster R4 and R5 that decreased at the end in Line1) in Line1 and Line2, the majority (62 non-synonymous mutations) should be “averaged” neutral mutations. Because of this neutrality, the hitchhiking events ensured a constant fixation rate of the non-synonymous mutations in the same manner as for synonymous mutations.

## Discussion

Under the severe selection pressure of thermal adaptation, we demonstrated that hitchhiking ran the molecular clock, despite the complicated dynamics of mutation fixation with the stochastic appearance of adaptive mutations and clonal inference. As neutral mutations accumulate on all genomes in a population spontaneously with equal probability, regardless of which genome gains an adaptive mutation to propagate or when, the fixation rate of the neutral mutations should coincide with the spontaneous mutation accumulation rate, suggesting that hitchhiking can be a mechanism for the molecular clock commonly observed in phylogenetic trees in addition to the genetic drift proposed by Kimura [23].

The effect of hitchhiking on molecular evolution rates has been investigated with sophisticated models and computer simulation [25,26]. In the present study, we proposed a simple model to elucidate the dependency of the hitchhike-driven molecular clock rate on the experimentally estimated parameters. To acquire an easy accessible and clear view on the dynamics of the molecular clock, we adopted only a few parameters to propose the model as follows. We classified the mutations in three categories, adaptive, harmful, and neutral, to avoid the complicacy of the epistasis between the mutations. The mutation that appeared lastly on a genome and dominated the population (*e*.*g*., > 10%) was categorized as the adaptive mutation. In the present case, the *cyaA* mutation was the adaptive mutation. As shown in Fig 3, the *cyaA* mutation positively contributed to the growth fitness of CloneB. It determinatively triggered the fixation of the other four mutations (*mutH*, *nadR*, *phoU/bglG* and *helD*, as Clone_1/94) and the domination of the population. In comparison, although the *helD* mutation positively contributed to the growth fitness when it appeared alone on CloneA, it was not the adaptive mutation but was categorized as the neutral mutation, as same as the rest three mutations. Thus, in this model, the neutral mutation could be either deleterious or beneficial when solely appeared but must be irrelevant at dominating the population when appeared with other mutations simultaneously on the identical genome. In addition to this cancelling effect between the beneficial and deleterious mutations, weakened efficacy of selection on non-synonymous mutations might be involved, because of clonal interference [24,37]. The harmful mutation was the deleterious mutation finally disappeared in the population.

To understand how the three categories of mutations participate in the dynamics of the molecular clock, we firstly assumed that every genome spontaneously accumulated the neutral mutations at the following rate:

R_ Neutral_Genome =μL(1−α−β) (1)

where *R_Neutral_Genome* is the average number of neutral mutations per genome per generation; *μ* is the spontaneous mutation accumulation rate (per bp per generation); *L* is the length of the genome (bp); and *α* and *β* are the fractions of the adaptive and harmful mutations. Here, *α* corresponds to the fraction of the adaptive mutations that become dominated and is smaller than the probability of the beneficial mutation. Thus, the population gains adaptive mutations at the following rate:

R_Adaptive_Population=μLαN (2)

where *R_Adaptive_Population* is the number of adaptive mutations per generation in the population, and *N* is the population size (the number of genomes in a prokaryotic population). The adaptive mutation mediated population domination takes place at the interval (generations) of the inverse of *R_Adaptive_Population* in average. The neutral mutations accumulate equally on each genome at the rate of *R_Neutral_Genome*, till an adaptive mutation appears and takes hitchhiking. Therefore, the average number of neutral mutations per hitchhiking (*N_Neutral_Hichhike*) is:

$$N\text{\_}Neutral\text{\_}Hitchhike\;=\frac{1}{\mu L\alpha N}\times \mu L(1-\alpha -\beta )=(1-\alpha -\beta )/\alpha N$$ (3)

As the adaptive mutation causes the fixation of the neutral mutations of *N_Neutral_Hichhike*, the number of the neutral mutations fixed in the population per generation (*R_Neutral_Population*) is:

R_Neutral_Population=μLαN×(1−α−β)/αN=μL(1−α−β) (4)

Consequently, the fixation rate of the neutral mutations by hitchhiking at the population level is equal to the spontaneous neutral mutation accumulation rate at the genome level, that is, *R_Neutral_Population* = *R_Neutral_Genome*. Noted that the fitness increase attributed to the adaptive mutations does not influence the rate of hitchhike-driven molecular clock of neutral mutations, according to the theoretical demonstration [25,26].

Based on the model, we depicted how the hitchhiking events occurred during the experimental evolution by estimating *α* and *β*. A total of 20 clusters of mutation accumulation (*i*.*e*., 20 genomes) were identified in the evolution. Note that the R4 and R5 clusters were excluded from the 22 clusters (S2 Table) in this analysis, because they interfered at the end of Line1. As these 20 genomes propagated independently for 4419 generations (5212 to 7580 in common, 7581 to 8829 in Line1, 7581 to 8382 in Line2), the adaptive mutations were supposed to occur at an interval of 221 generations in average, *i*.*e*., 1/*R_Adaptive_Population* = 221. If the synonymous mutations were all nearly neutral compared with the adaptive or harmful mutations at non-synonymous sites, the modification of *R_Neutral_Genome* for the neutral mutation accumulation rate for synonymous and non-synonymous sites turned to be as follows:

R_Neutral_Synonymous=μLs (5)

R_Neutral_Non-synonymous=μLn(1−α−β) (6)

where *Ls* and *Ln* are the total lengths of the synonymous and non-synonymous genomic sites. *R_Adaptive_Population* can be estimated with *μLnαN*, because the adaptive mutations are supposed to occur at the non-synonymous site. The average numbers of neutral mutations per hitchhiking (*N_Neutral_Hitchhike*) at the synonymous and non-synonymous sites are as follows:

R_Neutral_Synonymous/R_Adaptive_Population=Ls/LnαN (7)

R_Neutral_Non-synonymous/R_Adaptive_Population=(1−α−β)/αN (8)

Because 32 synonymous mutations were fixed through the propagation of the 20 genomes, the average number of neutral mutations on synonymous genomic sites per hitchhike *Ls*/*LnαN* was 1.6. With a population size *N* of 10^7^ and a ratio of synonymous sites over non-synonymous sites on the *E*. *coli* genome of *Ls*/*Ln* of 0.96 Mb/3.2 Mb = 0.3 [29], we estimated *α* as 2×10^−8^. The estimated value for the fraction of adaptive mutations *α* is small compared to previous estimates, even after considering clonal interference [33,38]. The small estimate occurs in part because only the adaptive mutations that triggered the hitchhikes are counted in *α*, while the other beneficial mutations that appeared and compensated for the deleterious mutations on the same genome were regarded as neutral in this study. In addition, as the 62 “averaged” neutral non-synonymous mutations became fixed during the 20 propagations, the average number of neutral mutations on non-synonymous genomic sites per hitchhike (1−*α*−*β*)/*αN* was 3.1, leading to an estimated *β* of 0.42. Approximately 42% of the mutations at non-synonymous sites were excluded from the population as harmful mutations, most likely due to the severe selection pressure from the successive temperature increments of 0.2°C. Based on the estimated values, the depicted dynamics for hitchhike occurrence were that the neutral mutation accumulation rates for synonymous and non-synonymous regions at the genomic level, *R_Neutral_Synonymous* = 0.01 and *R_Neutral_Non-synonymous* = 0.02, respectively, were faster than the adaptive mutation fixation rate at the population level, *R_Adaptive_Population* = 0.006. The excess accumulation of the neutral mutations hitchhiked on the rare adaptive mutations as theoretically proposed [25,26].

Regardless of the involvement of mutator phenotypes, the hitchhike-driven fixation rate for neutral mutations should coincide with the spontaneous mutation rate [25,26]. Obviously, the accelerated spontaneous mutation rate induced by the mutation of *mutH* resulted in the accumulation of many mutations in the genomes in the limited generations, resulting in the observation of the constant mutation fixation rates (Fig 5). Without the accelerated spontaneous mutation rate, we would have observed few neutral mutations, as we observed only one synonymous mutation until generation 5212 [29]. In long-term experimental evolution conducted by Lenski’s group, many synonymous mutations were observed after a mutator phenotype emerged [30]. The fixation rate of those synonymous mutations approximately coincided with the accelerated spontaneous mutation rate, suggesting that some of these mutations, if not all, might have been fixed by hitchhiking as observed in this study. These authors also reported that the synonymous mutations were not fixed before the appearance of the mutator, implying no hitchhike events. However, this result does not deny the independency of the hitchhike-driven fixation rate of neutral mutations from the mutator phenotypes. If the spontaneous mutation rate had remained without the two-order acceleration but at an ordinary value of 10^−10^/site/generation over 10^5^ generations, one could have observed the hitchhike-driven fixation of approximately ten synonymous mutations. Adaptive mutations also accumulated in a clock-wise manner in Lenski’s experimental evolution. The rate of adaptive mutation fixation has theoretically been investigated [39]. Long-term experimental evolutions would verify the molecular clock for neutral and adaptive mutations. As the coincidence of the fixation rate of neutral mutations at the population level with the spontaneous mutation accumulation rate at the genome level occurs in two fixation mechanisms, hitchhike and genetic drift, the dominant mechanism remains unclear. Genetic drift has been intensively investigated and has provided the theoretical backbone for the molecular clock of neutral evolution, in which organisms gain little fitness [23]. As the fixation of neutral mutations are supposed to be governed by genetic drift before the adaptive mutations take hitchhiking, it would be worthwhile to determine under what conditions genetic drift works to fix neutral mutations without the disturbance by hitchhiking in adaptive evolution.

Here, we discuss the question with a simple model which requires only the parameters that were estimated above, although there are sophisticated investigations on the fixation process of neutral mutations [12,27]. First, suppose that a neutral mutation appears on a genome in a population of size *N* and propagates through genetic drift, which requires many generations of typically the order of *N* for asexual organisms. To avoid sweeping out or fixing the neutral mutation by hitchhiking in its drifting process, no adaptive mutations should occur during *N* generations. The probability that no adaptive mutation occurs in a population of size *N* for *N* generations or longer is (1−*μLNα*)^*N*^. In the case of the evolution experiment in this study, the probability is virtually zero with the estimated parameters *μ* = 10^−8^, *L* = 0.32×10^7^, *α* = 2×10^−8^, *N* = 10^5^. In other words, for a neutral mutation to be fixed by genetic drift at a 50% probability (1−*μLNα*)^*N*^ = 0.5 or higher, the population size should be approximately on the order of 10^4^ or less. If one applies to the equation the typical spontaneous mutation rate of 10^−10^ for the non-mutator phenotype but an adaptive mutation fraction *α* of 2×10^−6^ because of the relief from clonal interference, the same population size of 10^4^ will be obtained. To apply the equation to eukaryotes, if the effective genome length L for adaptive mutations is replaced with the inverse of the recombination rate (*i*.*e*., 10^−8^/site/generation) multiplied by the sparseness of the coding region or non-neutral sites (for instance, 0.01), the threshold will be 10^5^. A similar threshold value above which hitchhiking dominates over genetic drift to fix neutral mutations has been proposed for a different reason [12]. Because a significant fraction of species have a population size of greater than 10^4~5^, some if not all of the neutral mutations that were observed in the phylogenetic trees might have been fixed by hitchhiking [40].

We experimentally demonstrated that hitchhiking ensured that the molecular clock for neutral mutations in fitness-increasing evolution occurred at the same rate as expected for genetic drift in neutral or fitness-steady evolution. The extension of the molecular clock, which has been verified for neutral evolution, to adaptive evolutions can provide insights into the past environment. Genetic drift, which requires the suppression of adaptive mutations over a long generation time, has led us to suppose that the past environment was sufficiently steady that the fitness contributions of fixed mutations could quickly change from positive to neutral by diminishing returns, leading to fitness-steady or neutral evolution. However, if the hitchhike-driven molecular clock runs for neutral mutations, adaptive mutations may occur frequently. Thus, it is reasonable to propose that the past environment was not always so steady but underwent dynamic changes that allowed organisms to adaptively evolve according to the hitchhike-driven molecular clock.

## Materials and Methods

### Strains and generations

A previously evolved *E*. *coli* strain 45L[29] (DH1*ΔleuB*:: *(gfpuv5-km* ^*r*^)) was used for prolonged thermal adaptive evolution. The generations were calculated according to the growth datasets as summarized in S3 Table in the present study and previously reported in Table S5. The generations of 45L (the final population) and 45A (the intermediate population in which the fitness contributions of accumulating mutations shifted from positive to neutral) were 7580 and 5212 and were incorrectly reported as 7560 and 5191 in the previous study [29]. The corrected generations are consistent with the raw data in supplemental Table S5 of the previous study.

### Cell culture

Cell culture was performed as previously reported with minor modifications [29]. Culture temperatures of 44.8°C, 45.0°C, 45.2°C, 45.4°C, 45.6°C, 45.8°C and 46.0°C were confirmed using a Platinum Resistance Thermometer 5615 (Fluke). Serial transfers of Line1 and Line2 were performed daily by diluting the exponential phase culture with pre-warmed (37°C) fresh medium. The dilution rate was determined to maintain cell growth within the exponential phase, *i*.*e*., the cell concentration was controlled at OD_600_ = 0.05–0.2 after 24 h of culture based on the growth rate estimated the previous day. During the temperature increase periods and the first one or two days of the culture restarted from the glycerol stock, the cell cultures were usually kept at a relatively high concentration, *e*.*g*., OD_600_>0.5. If the OD_600_ of the 24-h culture was lower than 0.05, the cell culture was extended for 24 h. The growth rate was calculated as described previously [29], and the details are summarized in S3 Table.

### Thermal niche analysis

Cells were inoculated from a glycerol stock and cultured at the corresponding adaptive temperature (44.8°C and 46.0°C, respectively). Following pre-cultures for 24 h, the cell cultures were transferred to fresh medium and incubated at 20.0°C, 37.0°C, 45.0°C, 46.0°C, 46.5°C and 47.0°C for 24 h. The growth rate at each culture temperature was calculated as the average value of triplicate cultures.

### Fluctuation test

The spontaneous mutation rate was determined by the fluctuation test as previously described [29]. The initial and final cell concentrations in all tests were controlled at approximately 1,000 and 5×10^7^−1×10^8^ cells/mL per tube, respectively. The cell concentrations were measured by flow cytometry (FACSCalibur, Becton, Dickinson and Company). A total of 35 tubes of 5 mL culture were used for each test. The cells in the culture were collected and transferred to plates containing 25 μg/mL streptomycin. After two days of incubation at 37°C, the numbers of plates without colonies were counted. The resultant probability, that is, the ratio of the number of plates without colony formation to the total number of plates, was used to calculate the spontaneous mutation rate.

### Genome resequencing and mutation analysis

The cell populations at the beginning of prolonged evolution and at the ends of Line1 and Line2 were subjected to next-generation resequencing analyses. Cells grown until stationary phase were collected as previously described [29]. Genomic DNA was purified and fragmented using the Covaris system as previously described [41]. Whole-genome resequencing was performed using the Applied Biosystems SOLiD 3 system according to the manufacturers’ instructions. Replicates of 50-base mate pair libraries (two quarters of a slide) were prepared for each sample. The overall procedure of library preparation, resequencing reaction and base calling was performed according to the manufacturer’s recommendations. The sequence reads were assembled and aligned using Bioscope v2.0 (Applied Biosystems). The reads in each dataset were mapped to the ancestor genome *DH1* [41], to acquire the results of single nucleotide substitutions. The further confirmation of the detected mutations, the temporal change and heterogeneity of the mutations in the cell populations were performed using the Sanger method, as described in the following section.

### Population analysis of mutation fixation

All single nucleotide substitution mutations identified by genome resequencing were further confirmed by Sanger methods. The mutation fixation dynamics were evaluated by Sanger sequencing of the stocked cell populations that were acquired in the evolution experiments. The cell stocks were directly subjected to Sanger sequencing to avoid the possible biased enrichment of heterogeneity within the populations. The results of the Sanger sequences were visualized using the software VectorNTI (Invitrogen) or 4Peaks (Mek&Tosj). The changes in mutation fixation dynamics were determined according to the ratio of the peak values representing the wild type and the substituted nucleotides in each population for each mutation. The analyses and graphics of the mutation fixation dynamics were generated using the software Mathematica v9.0 (Wolfram).

### Single-cell isolation

Single-cell isolation of the cells that were stocked during the evolution experiments was performed to purify the genotypes among the populations. The glycerol stocked cells were precultured at 44.8°C, and the cell concentrations of the cultures were determined by flow cytometry (FACSCalibur, Becton, Dickinson and Company). The cell populations were subsequently diluted with fresh media and inoculated into 96-deep-well plates at concentrations of 0.1 cells/well in 200 μL per well. The plates were incubated at 37°C with shaking at 1,100 rpm (Deepwell Maximizer MBR-022UP, TAITEC) for two or three days. The wells that became turbid were a result of single-cell-derived growth. The cells were checked for growth at 44.8°C and finally harvested for further mutation analyses.

## Supporting Information

S1 Fig Standard curve for estimating the mutant frequency in a population from the peak height of Sanger sequencing. The two clones that were isolated from the population at generation 6448, one with all 10 of the substitutions in *atoE*, *cyaA*, *lldP*, *putA*, *rihB*, *yehB*, *ygfO*, *yidD*, *ydaM*, and *nrdF* in the fourth cluster, A4 (see S2 Table) and the other with none of them, were mixed at various ratios before Sanger sequencing. The relative peak heights of the original and substituted bases were determined for all 10 sites to obtain the average and standard error of the ratios.
(TIF)

S1 Table Newly identified mutations in Line1 and Line2. The Gene names, JWID, position in DH1 are from GenoBase. The Position, Codon change, amino acid change of each genome mutation are listed with the functional descriptions of the corresponding gene.
(XLS)

S2 Table Temporal changes in the frequencies of the clustered mutations. The frequencies of each genomic mutation measured at the indicated generations from the middle of the previous adaptive evolution (Kishimoto et al 2010) to the ends of the following Line1 and Line2. The temperature at each generation was listed. The cluster No, JWID and gene name of each genomic mutation are indicated with description on synonymous or non-synonymous mutation in the column (S / N).
(XLS)

S3 Table Temporal changes in the specific growth rates of Line1 and Line2. Generation was calculated based on the increase in OD as described in Material and Method section. Cumulative generation started at generation 7580, at which the previous adaptive evolution (Kishimoto, et al. 2010) was duplicated and elongated to Line1 (left) and Line2 (right).
(XLS)
