# Supplementary figures and images for: Molecular Clock of Neutral Mutations in a Fitness-Increasing Evolutionary Process
Source: PLoS Genet. 2015 Jul 15;11(7):e1005392. doi: 10.1371/journal.pgen.1005392 (PMC4503671; doi:10.1371/journal.pgen.1005392)

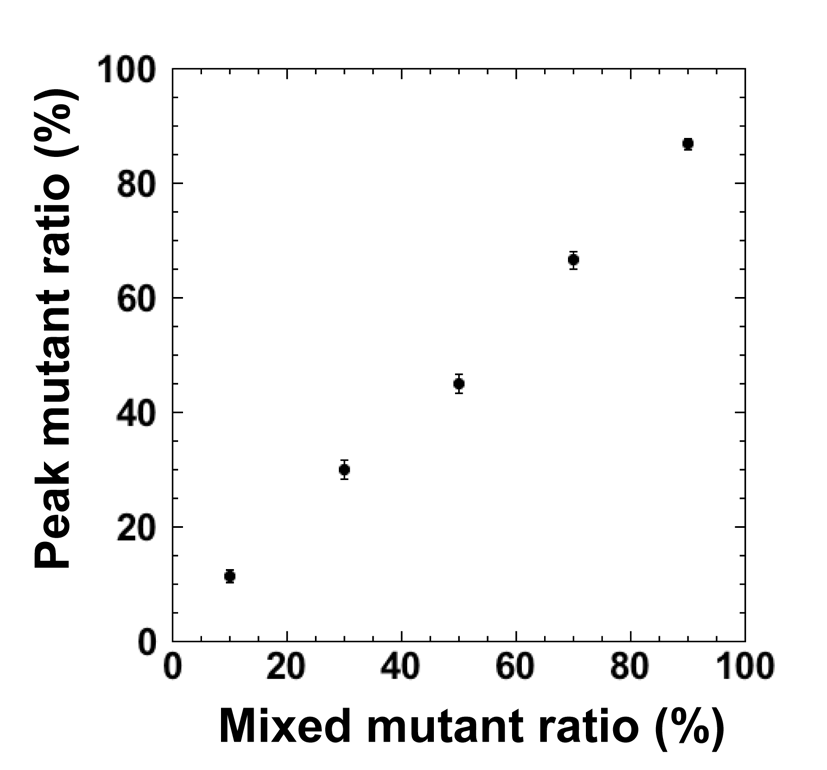

Supplement: S1 Fig — The two clones that were isolated from the population at generation 6448, one with all 10 of the substitutions in atoE, cyaA, lldP, putA, rihB, yehB, ygfO, yidD, ydaM, and nrdF in the fourth cluster, A4 (see S2 Table) and the other with none of them, were mixed at various ratios before Sanger sequencing. The relative peak heights of the original and substituted bases were determined for all 10 sites to obtain the average and standard error of the ratios. (TIF) [file pgen.1005392.s001.tif]
